# ABCD1 dysfunction alters white matter microvascular perfusion

**DOI:** 10.1093/brain/awx262

**Published:** 2017-11-09

**Authors:** Arne Lauer, Xiao Da, Mikkel Bo Hansen, Gregoire Boulouis, Yangming Ou, Xuezhu Cai, Afonso Liberato Celso Pedrotti, Jayashree Kalpathy-Cramer, Paul Caruso, Douglas L Hayden, Natalia Rost, Kim Mouridsen, Florian S Eichler, Bruce Rosen, Patricia L Musolino

**Affiliations:** 1 Department of Neurology, Massachusetts General Hospital, Boston, MA, USA; 2 Department of Neuroradiology, Goethe University, Frankfurt a.M., Germany; 3 Athinoula A. Martinos Center for Biomedical Imaging, Charlestown, MA, USA; 4 Department of Clinical Medicine, Aarhus University, Denmark; 5 Department of Neuroradiology, Université Paris-Descartes, INSERM UMR 894, Centre Hospitalier Sainte-Anne, Paris, France; 6 Fetal-Neonatal Neuroimaging and Developmental Science Center, Boston Children’s Hospital, Boston, MA, USA; 7 Department of Radiology, Massachusetts General Hospital, Boston, MA, USA; 8 Department of Biostatistics, Massachusetts General Hospital, Boston, MA, USA

**Keywords:** cerebral X-linked adrenoleukodystrophy, ALD, ABCD1, inflammatory demyelination, microvascular perfusion

## Abstract

Cerebral X-linked adrenoleukodystrophy is a devastating neurodegenerative disorder caused by mutations in the *ABCD1* gene, which lead to a rapidly progressive cerebral inflammatory demyelination in up to 60% of affected males. Selective brain endothelial dysfunction and increased permeability of the blood–brain barrier suggest that white matter microvascular dysfunction contributes to the conversion to cerebral disease. Applying a vascular model to conventional dynamic susceptibility contrast magnetic resonance perfusion imaging, we demonstrate that lack of ABCD1 function causes increased capillary flow heterogeneity in asymptomatic hemizygotes predominantly in the white matter regions and developmental stages with the highest probability for conversion to cerebral disease. In subjects with ongoing inflammatory demyelination we observed a sequence of increased capillary flow heterogeneity followed by blood–brain barrier permeability changes in the perilesional white matter, which predicts lesion progression. These white matter microvascular alterations normalize within 1 year after treatment with haematopoietic stem cell transplantation. For the first time *in vivo*, our studies unveil a model to assess how ABCD1 alters white matter microvascular function and explores its potential as an earlier biomarker for monitoring disease progression and response to treatment.

## Teaser

X-linked adrenoleukodystrophy is a neurodegenerative disorder caused by mutations in *ABCD1*. By applying a vascular model to conventional MR perfusion imaging, Lauer *et al.* demonstrate that ABCD1 deficiency causes alterations in microvascular flow in white matter areas and developmental stages with the highest probability for conversion to cerebral disease.

## Introduction

**Figure 1:**
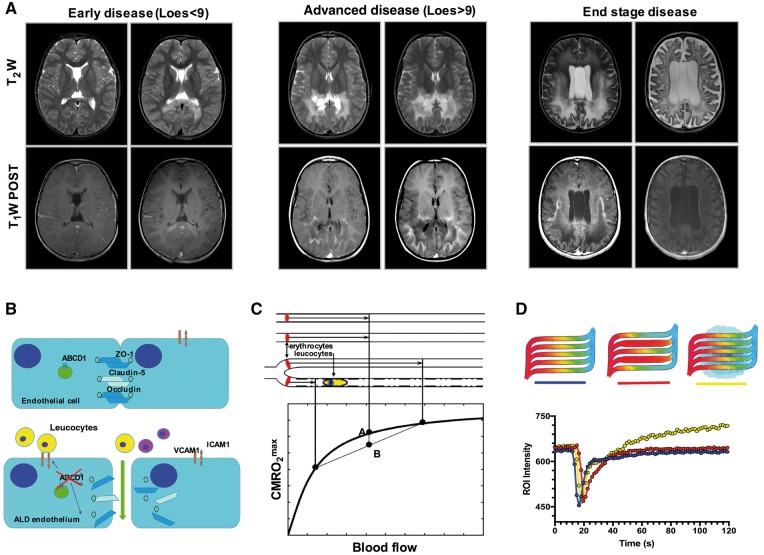
**Cerebral adrenoleukodystrophy and proposed effects of ABCD1 deficiency upon brain microvascular system.** (**A**) Representative T_2_-weighted (T_2_W) and T_1_-weighted (T_1_W) post-contrast images at baseline (*left*) and follow-up (*right*) in early (Loes score: 1 and 3), advanced (Loes score: 18 and 23) and end stage (Loes score: 20 and 30) of cerebral adrenoleukodystrophy (CALD). These cases illustrate the stereotypical symmetrical contiguous spread of T_2_-weighted-hyperintensity during lesion progression (*upper* panels) and the rim of active inflammation as indicated by contrast enhancement on T_1_-weighted images (*lower* panels). (**B**) Schematic illustration of the effects of ABCD1 deficiency upon endothelial function depicting increased adhesion of blood born leucocytes and increased blood–brain barrier permeability. (**C**) Vascular model applied to analyse tracer kinetics of dynamic susceptibility contrast perfusion MRI data. According to this model, when capillaries fail to homogenize their mean transit flow times (elevated CTH), as when slow leucocyte transit occurs, their tissue OEC and therefore the theoretical upper limit of metabolic rate of oxygen (CMRO_2_^max^) is compromised. (**D**) Schematic of flow dynamics across capillary beds where homogenous, heterogeneous and leaky microvasculature are represented. Graphed intensity curves demonstrating elevated microvascular flow heterogeneity (CTH, red) and exacerbated blood–brain barrier permeability (K_app_, yellow). ROI = region of interest.

X-linked adrenoleukodystrophy (ALD) is caused by mutations in the *ABCD1* gene that lead to accumulation of very long chain fatty acids (VLCFA) in plasma and tissues (Mosser *et al.*, 1993; Berger *et al.*, 2014). Loss of function of the *ABCD1* gene can cause different phenotypes ranging from asymptomatic to adrenal insufficiency, adrenomyeloneuropathy (AMN) and an aggressive form of cerebral inflammatory demyelination (CALD) that leads to vegetative state or death within 2–5 years (Fig. 1A) (van Geel *et al.*, 2001; Moser *et al.*, 2005; Berger *et al.*, 2014; Kemp *et al.*, 2016). It is clear that other factors (‘hits’) modulate the conversion of phenotype since the ABCD1 mutation is necessary but not sufficient to develop cerebral disease. The risk of suffering symptoms for most phenotypes increases with age (Kemp *et al.*, 2016) but CALD is much more likely to occur during childhood and is rare beyond adolescence (Eichler *et al.*, 2007; Moser *et al.*, 2007), challenging the accepted notion that pathogenic processes observed in ALD are related to progressive toxic accumulation of VLCFA (Berger *et al.*, 2014; Kemp *et al.*, 2016). Recent *in vitro* work suggests indeed that lack of ABCD1 causes direct brain endothelial dysfunction preceding the accumulation of VLCFA and leading to higher interactions between endothelium and leucocytes (Musolino *et al.*, 2015; Kemp *et al.*, 2016). During inflammatory states leucocytes diminished deformability and increased adhesion to activated endothelium as they passage through individual capillaries can alter microvascular flow dynamics and tissue perfusion (Fig. 1B). (Schmid-Schönbein *et al.*, 2001; Aubourg, 2015). Evidence of abnormal microvascular function in ALD patients includes: (i) *ex vivo* histopathology showing distorted microvascular permeability beyond the edge of the demyelinating lesions (Musolino *et al.*, 2015); (ii) *in vitro* experiments showing that lack of ABCD1 in human brain microvascular endothelial cell causes increased adhesion and permeability to leucocytes (Musolino *et al.*, 2015); and (iii) *in vivo* MRI demonstrating that contrast extravasation (Loes *et al.*, 2003) and white matter hypoperfusion predict lesion progression and clinical outcome after haematopoietic stem cell transplantation (HSCT) (Musolino *et al.*, 2012; McKinney *et al.*, 2016). Magnetic resonance perfusion abnormalities have also been found to be associated with white matter lesion progression in other inflammatory demyelinating disorders, such as multiple sclerosis, optic neuritis, acute disseminated encephalomyelitis and progressive multifocal leukencephalopathy (Khoury *et al.*, 2013; Cramer *et al.*, 2015; D'haeseleer *et al.*, 2015; Hiremath *et al.*, 2017).

The lack of adequate experimental models represents a major obstacle to understanding whether microvascular dysfunction and increased blood–brain barrier permeability are secondary to ongoing myelin degeneration or are themselves consequences of the lack of ABCD1 function and a trigger of cerebral disease. Transgenic mice do not exhibit inflammatory demyelination (Forss-Petter *et al.*, 1997; Lu *et al.*, 1997; Pujol *et al.*, 2004) and autopsy studies are limited to advanced stages of CALD (Schaumburg *et al.*, 1975; Eichler *et al.*, 2008; van der Voorn *et al.*, 2011; Musolino *et al.*, 2015). Moreover, even though conventional brain MRI is exquisitely sensitive in detecting cerebral disease months before the onset of neurological symptoms, it does not predict conversion to CALD or early lesion progression (Moser *et al.*, 2000; Eichler *et al.*, 2002; Loes *et al.*, 2003; Ratai *et al.*, 2008). As uniform newborn screening is implemented in all states in the USA and other countries (Kemper *et al.*, 2016) a biomarker that determines which patients are at high risk of CALD is urgently needed.

In this study, we apply a vascular model to dynamic susceptibility contrast (DSC) magnetic resonance perfusion acquired in an ALD patient cohort followed at our academic medical centre over a period of 10 years (Supplementary Table 1) to estimate capillary transit time heterogeneity (CTH), a marker of microvascular flow dynamics; and the apparent contrast leakage constant (K_app_) as an indicator of blood–brain barrier permeability (Fig. 1B) (Mouridsen *et al.*, 2006, 2014; Bjornerud *et al.*, 2011; Jespersen and Østergaard, 2012; Hansen *et al.*, 2017). Given that regulation of flow across a capillary bed steers oxygen availability to match metabolic demands, our model also allowed us to examine metabolic parameters including oxygen extraction capacity (OEC) and the upper limits of metabolic rates of oxygen (CMRO_2_^max^) (Jespersen and Østergaard, 2012).

The purpose of this study is to provide a first *in vivo* assessment of white matter microvascular physiology over time in ALD hemizygote patients with and without CALD. To determine the temporal and anatomical associations between CTH, K_app_, OEC and CMRO_2_^max^ with disease onset, lesion progression and response to treatment we examined affected and unaffected white matter regions of ALD subjects at different time points and compared them with unaffected white matter and age-matched control subjects. Three different mixed-effects models were constructed to estimate how perfusion parameters relate to age, anatomical location and volume lesion growth over time.

## Materials and methods

### Subjects

Overall, 45 male subjects with ALD (CALD, self-arrested CALD and hemizygotes without signs of CALD) were evaluated at Massachusetts General Hospital between January 2006 and July 2016 (Supplementary Table 1). MRI was performed for clinical monitoring of disease progression. Dynamic susceptibility contrast magnetic resonance perfusion imaging (DSC-MRI) was acquired in 40 subjects. We included 25 age-matched subjects, identified by a keyword search, who underwent magnetic resonance perfusion imaging during routine clinical care and showed no evidence of perfusion or structural abnormalities within the regions of interest as control subjects. Younger children and symptomatic subjects required general anaesthesia, while asymptomatic subjects older than 7 years underwent magnetic resonance scanning without sedation. The study received ethical approval by the Institutional Review Board of MGH (MGH protocol 2012-P-000132/1).

### Structural MRI

The magnetic resonance studies were performed on two magnetic resonance scanners: a 1.5 T GE Signa HDx, GE healthcare with an eight-channel head coil, and a 3.0 T MAGNETOM TioTrim, Siemens equipped with a 12-channel head coil. The magnetic resonance protocol included an axial T_2_-weighted sequence (repetition time/echo time/slice thickness, matrix, field of view in 1.5 T: 5350–7850 ms/95–112 ms/3–4 mm/512 × 512/22 × 22–24 × 24 cm; and in 3.0 T: 4200–9000 ms/97–143 ms/3–4 mm/480–512 × 512/22 × 22–24 × 24 cm), pre- and post-contrast axial T_1_-weighted spin-echo sequences (repetition time/echo time/slice thickness/matrix/field of view in 1.5 T: 450–600/14–20 ms/3–4 mm/256 × 256/22 × 22–24 × 24 cm; in 3.0 T: 500–742 ms/1.74–9.1 ms/3.0 mm/480–512 × 512/24 × 24 cm) and axial diffusion tensor imaging (DTI; repetition time/echo time/slice thickness/matrix/field of view/number of directions in 1.5 T: 10 000 ms/87–99 ms/2.2–2.4 mm/256 × 256/22 × 22 cm/25; and in 3.0 T: 3800–9700 ms/90–92 ms/2.0–5.0 mm/160 × 160/23 × 23 cm/25).

### Magnetic resonance perfusion imaging

Dynamic susceptibility contrast magnetic resonance perfusion imaging (DSC-MRI) was performed for all subjects using gradient echo (GRE) echo planar imaging (EPI) sequences. Acquisition parameters were repetition time/slice thickness/matrix/field of view: 1500 ms/40 ms/5 mm/128 × 128/22 × 22 cm for both 1.5 T and 3 T scanners. The echo time was 40 ms and 32 ms at 1.5 T and 3 T scanners, respectively. A total of 60 (1.5 T) or 80 (3 T) dynamic acquisitions were acquired before, during, and after injection of 0.1 mM/kg gadolinium-based contrast [gadolinium diethylenetriamine pentaacetic acid (Gd-DTPA), Bayer Schering]. Contrast was injected at 5 ml/s for older subjects using a power injector, while the injection was performed manually for the paediatric subjects using maximal permissible rates depending on their intravenous access (up to 5 ml/s). Incomplete magnetic resonance perfusion datasets or those significantly degraded by motion artefacts were excluded from the analysis.

### Image processing

#### Quantification of CTH, OEC, CMRO_2_^max^ and blood–brain barrier permeability

The included DSC perfusion magnetic resonance datasets were postprocessed using Penguin perfusion software (www.cfin.au.dk/software/penguin) (Mouridsen *et al.*, 2014). Perfusion maps were calculated using deconvolution with an automatically selected arterial input function (Mouridsen *et al.*, 2006). Capillary transit time heterogeneity (CTH) was estimated as the standard deviation of a model transit time distribution obtained as a part of voxel-wise fitting of a vascular model to individual concentration-time curves obtained from the DSC MRI data:

(1) $$\kappa c(t)=CBF\int_{0}^{t}C_{a}(\tau )h(t-\tau |\alpha ,\beta )d\tau +K_{app}\int_{0}^{t}C_{a}(\tau )d\tau$$

where, in the first term, CBF is the cerebral blood flow, C_a_(t) the arterial input function, h(t) the residue function. The second term represents possible tracer extravasation quantified by the apparent leakage parameter K_app_ (Hansen *et al.*, 2017). κ is a constant dependent on the haematocrit in the arterioles and capillaries, and the density of brain tissue, but is assumed constant and set to unity. In Equation 1, the residue function represents the fraction of tracer still present in the capillaries at a time *t* after injection and is interpreted as the complement of a cumulative gamma distribution, (Mouridsen *et al.*, 2006, 2014) where the gamma distribution is parametrized as:

(2) $$h(t|\alpha ,\beta )=\frac{1}{\beta^{\alpha }\Gamma (\alpha )}\int_{0}^{t}\tau^{\alpha -1}e^{-\tau /\beta }d\tau$$

where α and β are model parameters and Γ denotes the gamma function.

We estimated extraction of oxygen as described in Jespersen and Østergaard (2012). Briefly, passive diffusion of oxygen along a single capillary with fixed transit time may be computed using the Bohr-Kety-Crone-Renkin model (Renkin, 1985). Then, as the transit times vary throughout a capillary network, we use the estimated distribution of transit times in Equation 1 to calculate the voxel-wise total oxygen extraction capacity (Jespersen and Østergaard, 2012):

(3) $$OEC=\int_{0}^{\infty }Q(\tau )h(\tau |\alpha ,\beta )d\tau$$

where Q(τ) is the oxygen extraction along a single capillary with transit time τ.

The upper limit of metabolic rate of oxygen (CMRO_2_^max^) was calculated based on the relationship of OEC and CBF (Jespersen and Østergaard, 2012);

(4) $$CMRO_{2}^{\max}=CBF\cdot OEC$$

Extravasation of contrast agent was quantified to generate the blood–brain barrier permeability maps from the tissue residue function in DSC MR Perfusion data as previously described (Bjornerud *et al.*, 2011). We note that the leakage correction part of the vascular model was slightly modified compared to Bjornerud *et al.* (2011) to obtain an operational model for describing CTH, OEC, and CMRO_2_^max^ in the potential presence of contrast agent leakage (Hansen *et al.*, 2017).

#### Image co-registration

All images were converted into the NIfTI image format and corrected for magnetic field inhomogeneities (Tustison *et al.*, 2010). Perfusion and diffusion maps were co-registered to structural MRI images using an attribute-based image registration algorithm (DRAMMS, http://www.rad.upenn.edu/sbia/software/dramms/index.html) (Ou *et al.*, 2011). Longitudinal data in the same subjects were registered affine to baseline scans.

### Quantification of whole white matter perfusion

Whole white matter perfusion was analysed in 10 hemizygote subjects without evidence of CALD (hemizygote subjects) and 10 age- and scanning protocol matched male controls (controls, Supplementary Table 2). Haematocrit levels and anaesthesia records in the children were reviewed to avoid confounding factors. Individual T_1_-weighted sequences were skull stripped, non-uniformity corrected and registered to the same structural MRI space as described above (Tustison *et al.*, 2010; Ou *et al.*, 2011). The T_1_-weighted sequence was classified into grey matter, white matter and CSF with an automated classification algorithm (Zhang *et al.*, 2001). Co-registered perfusion-based maps were masked with white matter segmentation results and statistical data were derived from the selected regions.

### Atlas of spatial cerebral lesion distribution

Binary lesion masks of 35 subjects were created with MRIcron (https://www.nitrc.org/projects/mricron) using an adopted semi-automated, multistep protocol to quantify white matter lesion burden (defined as T_2_-weighted hyperintense signal abnormalities) on the first available axial T_2_-weighted sequences of each CALD patient (Chen *et al.*, 2006). Individual T_2_-weighted maps were corrected for magnetic field inhomogeneities, co-registered as described above, and mapped into a common atlas space using deformable registration (Ou *et al.*, 2011). The same deformations were used to transform the annotated binary lesions masks from each individual’s space to the atlas space (Akbari *et al.*, 2014). Transformed binary lesion masks were superimposed and normalized by the number of subjects to create the lesion probability map in the atlas space.

### Lesion segmentation and regions of interest

In subjects with CALD, five different concentric zones based on abnormal T_2_-weighted and T_1_-weighted-post-contrast signals were selected: Zone A (central, T_2_-weighted hyperintense, non-enhancing), Zone B (T_2_-weighted hyperintense, with abnormal enhancement), Zone C (T_2_-weighted hyperintense, non-enhancing), Zone D [normal-appearing white matter (NAWM) adjacent to the lesion] and Zone E [distant NAWM (dNAWM)]. Infratentorial and corticospinal tract lesions were excluded. 3D Slicer (http://www.slicer.org/) was used to place regions of interest bilaterally in each zone (equivalent to 24 voxels on perfusion maps) blinded to perfusion data by two independent readers [A.L. and G.B.; intra-class correlation coefficient (ICC) for CTH = 0.84]. In all subjects, including those without brain lesions (controls, hemizygotes), nine additional regions of interest within the white matter (Fig. 3A) and one within the thalamus were selected in the same fashion for normalization purposes and longitudinal studies. Further, volumetric regions of interest were drawn based on co-registered T_2_-weighted or T_1_-weighted post-contrast maps. Longitudinal data from 10 subjects without interval HSCT were included in all combined analyses. To investigate perfusion in adjacent NAWM converting to T_2_-weighted-hyperintense lesion on follow-up, volumetric regions of interest were drawn radially beyond the lesion edge defined as T_2_-weighted-hyperintense on baseline (Zone D, as defined above). Regions appearing normal on baseline, but T_2_-weighted-hyperintense on follow-up (Zone C) were subtracted to be compared to regions remaining normal appearing (Fig. 5B). Second, T_2_-weighted-hyperintense, non-enhancing on T_1_-weighted regions (Zone C) were divided into enhancing versus non-enhancing on T_1_-weighted follow-up in the same fashion (Fig. 5C). Lesion delineation was performed using MRIcron as described above, blinded to perfusion maps (A.L.). Measurements were repeated by a second reader (G.B.) with consistent results (ICC = 0.93 for T_2_-weighted lesion progression and CTH; ICC = 0.90 for T_1_-weighted post-contrast lesion and K_app_ values).

### Evaluation of structural MRI

The severity of cerebral abnormalities was assessed by two experienced readers (A.L.C.P., P.C.) on sagittal T_1_-weighted- and axial T_2_-weighted brain MRI scans using the adrenoleukodystrophy MRI Loes scale (34-point scale for quantification of white matter abnormalities in the cerebrum, brainstem and cerebellum) (Loes *et al.*, 1994). A consensus meeting was performed involving a third reader (P.L.M.) in cases of disagreement. Untreated ALD presenting with a CALD consistent lesion without contrast enhancement on T_1_-weighted maps and lesion progression in the observation period of at least 1 year were defined as self-arrested. The investigators who performed the MRI scoring were blinded to the results of the magnetic resonance perfusion analyses and maps as well as treatment status.

### Statistical analysis

Assuming a two-sided α-level of 0.05 and homogeneous variances for the samples to be compared we calculated 80.0% power to detect effect sizes of 0.19 to 0.12 for the numbers of available subjects by using the means and common standard deviation for CTH and K_app_ from pilot data. Shapiro-Wilk test was used to test for normality. In subjects with cerebral disease mean perfusion (CTH, K_app_, OEC, CMRO_2_) in the five Zones A–D, and dNAWM were evaluated using one-way ANOVA with repeated measures followed by a Dunnett’s multiple comparisons test and mean DTI [ADC (FA, B0; not shown)], mean T_2_-weighted and T_1_-weighted post-contrast signal intensity values using Friedman test with Dunn’s corrections for multiple comparisons. Paired Student’s *t*-test was used to compare mean values of regions of interest within scans. Unpaired Student’s *t*-test was used to compare perfusion values between groups. For temporal analysis of microvascular flow pattern in the splenium of the corpus callosum the CTH was normalized to individual’s thalamus perfusion [relative (r)CTH] and plotted against age. In contrast to hemizygotes, no follow-up imaging was available in controls. To investigate effects of HSCT on perfusion over time, perfusion values in Zones C and D normalized to dNAWM were compared to pretreatment in subjects that underwent HSCT.

Three different mixed-effects models were constructed to investigate how perfusion parameters relate to age, anatomical location and volume lesion progression over time, using Proc Mixed procedure in SAS 9.4 (SAS Institute, Cary, NC). We decomposed age into two uncorrelated components: (i) each subject’s mean age over all the ages at which that subject has been observed; and (ii) each subject’s change in age calculated as the difference between each observed age and the subject’s mean age. The mean age accounts for the between-subject effect of increasing age while the change in age accounts for the within-subject effect of increasing age, which could vary from subject to subject. Given that the field strength of the scanner platform affects lesion volume quantification and perfusion parameter measurements, the interactions with the scanning protocols were evaluated as fixed effects. Simplified models without these terms are presented in this manuscript after the testing did not reveal significant interactions within the models. We report regression coefficients as β, the change in the dependent variable per unit change in the independent variable controlling for all other variables in the regression models.

- Model 1: To evaluate the association of CTH with the probability of demyelinating lesion appearance in different white matter regions controlling for age, a random effects model was used to regress CTH on fixed effects of probability, mean age, change in age, and a mean age × change in age interaction. The model includes a random intercept and random effects of change in age and probability for each subject to account for the correlation of repeated observations within each subject**.**
- Model 2: To evaluate the association of the log of rCTH with age and phenotype a random effects model was used to regress log rCTH on the fixed effects of mean age, phenotype, and a mean age × phenotype interaction. The model includes a random intercept for each subject to account for the correlation of repeated observations within each subject.
- Model 3: To evaluate the association of the log of lesion volume with CTH, age, and treatment, a random effects model was used to regress log lesion volume on fixed effects of mean age, change in age, treatment, flow heterogeneity in perilesional NAWM on the previous scan, and the interaction between mean age and change in age, change in age and treatment, and treatment and flow heterogeneity in perilesional NAWM on the previous scan. The model includes a random intercept and random effect of change in age for each subject to account for the correlation of repeated observations within each subject.

## Results

### ABCD1 deficiency alters white matter microvascular perfusion even in the absence of cerebral disease

**Figure 2:**
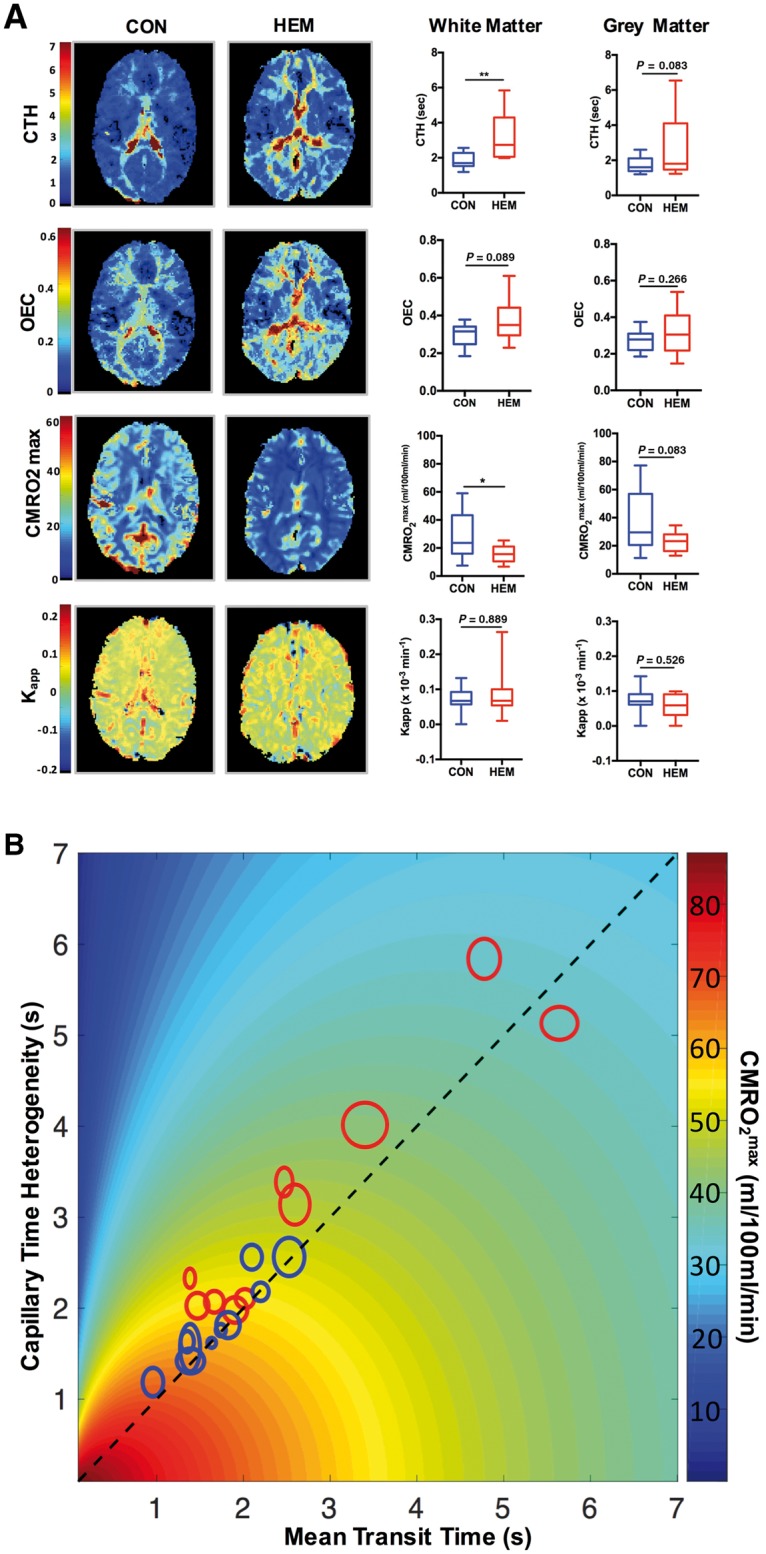
**ABCD1-related changes in microvascular perfusion in ALD subjects without cerebral disease**. (**A**) Representative CTH, OEC, CMRO_2_^max^ and K_app_ maps of a 14-year-old male control (CON) and a 16-year-old male hemizygote without CALD (HEM) and group comparisons controls versus hemizygote subjects. Data are presented as Tukey plots and are representative of 10 matched controls and 10 hemizygote cases. *P < 0.05, **P < 0.01, two-tailed *t*-test. (**B**) Diagram displaying individual white matter perfusion mean values and standard deviation (proportional to circle size) in relationship to CTH, mean transit time (MTT) and CMRO_2_^max^ in hemizygote subjects and controls. Deviation from the 45° line indicates a mismatch in mean transit time and CTH.

We examined 10 asymptomatic ALD subjects (hemizygotes) and found higher values of CTH in whole white matter when compared to age and sex-matched controls (3.20 ± 0.44 s versus 1.83 ± 0.15 s; unpaired samples *t*-test, *P* < 0.01, Fig. 2A). Based on the same raw perfusion data we also observed reduced O_2_ availability as shown by lower CMRO_2_^max^ values in the white matter of hemizygote subjects despite a tendency to higher OEC as compared to controls (15.78 ± 1.97 ml/100 ml/min versus 29.18 ± 5.58 ml/100 ml/min; unpaired samples *t*-test, *P* = 0.04) (Fig. 2A). A similar but not statistically significant pattern was observed in the grey matter of hemizygotes. Subjects without inflammatory demyelinating lesions leakage terms were not found to be different between hemizygotes and controls (K_app_: 0.08 ± 0.03 × 10^−3^/min versus 0.07 ± 0.01 × 10^−3^/min; unpaired samples *t*-test, *P* = 0.84, Fig. 2A). Further white matter perfusion in hemizygotes appears to display a mismatch between mean transit time and CTH more frequently, indicating capillary deficiency on adjusting to changes in flow velocities, which ultimately leads to a shift towards lower oxygen availability (Fig. 2B).

### Spatial and temporal perfusion abnormalities are associated with probability of demyelination

**Figure 3:**
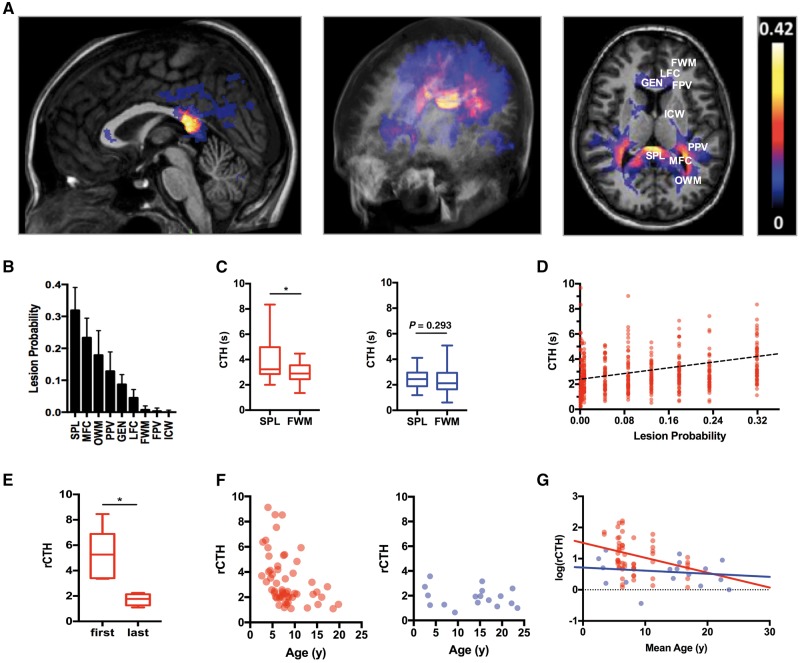
**Spatial and temporal microvascular perfusion abnormalities in ABCD1 deficiency.** (**A**) Statistical atlas of spatial lesion distribution based on first available MRI scans in 35 subjects with CALD. The probability map indicates percentage white matter involvement based on T_2_-weighted signal abnormalities. Axial images depict regions of interest as follows: SPL = splenium of the corpus callosum; MFC = major forceps; OWM = occipital white matter; PPV = posterior periventricular white matter; ICW = internal capsule white matter; FPV = frontal periventricular white matter; LFC = lesser forceps; GEN = genu of the corpus callosum and FWM = frontal white matter; and plot ranking the probabilities of being affected by CALD for each region of interest. (**B**) Comparison of capillary transit time heterogeneity (CTH) within the splenium of the corpus callosum versus frontal white matter in 10 hemizygote subjects (red) without cerebral disease and 18 controls (controls = blue). Data are presented as Tukey plots, of a paired two-tailed *t*-test, *P ≤ 0.05. (**C**) Comparison of longitudinal data of rCTH (rCTH = SPL CTH/thalamic CTH) for the first and last available scans in hemizygote subjects in absence of CALD. (**D**) Random effects modelling association between rCTH and lesion probability based on 51 scans of eight hemizygote subjects. Regression coefficient: β = 3.8452 ± 0.2695, *P* < 0.001. (**E**) Comparison of longitudinal data of rCTH in the SPL normalized to thalamic perfusion for the first and last available scans in hemizygote subjects in absence of CALD. (**F**) Individual rCTH values plotted against age for *n* = 8 hemizygote subjects (*left*) and *n* = 22 controls (*right*). (**G**) Diagram illustrating effect of age on rCTH applying a random effects model to compare hemizygote subjects and controls. Regression coefficients: hemizygote subjects: β = −0.05185, 95% CI −0.093 to −0.010, *P* = 0.0176; controls: β = −0.00948, 95% CI −0.047 to 0.028, *P* = 0.6048.

To assess if CTH changes observed in hemizygotes correlated with anatomical white matter regions commonly affected by inflammatory demyelination, we first calculated the disease probability for each anatomical white matter area based on a statistical atlas of lesion distribution derived from T_2_-weighted maps from 35 subjects with CALD (Fig. 3A and B). In hemizygote subjects, we found that the splenium of the corpus callosum, the most frequently affected region, carries the highest heterogeneity of flow (CTH: 4.00 ± 0.60 s) when compared to the less frequently affected white matter areas such as the frontal white matter (CTH: 2.92 ± 0.27 s, paired samples *t*-test, *P* = 0.039, *n* = 10). These white matter anatomical differences in CTH were absent in controls (2.43 ± 0.16 s versus 2.32 ± 0.23 s, paired samples *t*-test, *P* = 0.6231, *n* = 25, Fig. 3C). To evaluate the association of CTH with the probability of demyelinating lesion appearance for different white matter regions in hemizygotes, we applied a random effects model controlling for the effect of age upon CTH and found that CTH increases ∼1 unit (s) for each 25% absolute increase in probability of developing demyelination (β = 3.8452 ± 0.2695, *P* < 0.001, Fig. 3D and Model 1). To determine if age also influences CTH we compared the first and last available scans in hemizygote subjects and found that normalized capillary flow heterogeneity (rCTH) in the splenium of the corpus callosum decreases with age (rCTH: 5.17 ± 0.92 versus 1.7 ± 0.21, paired *t*-test, *P* = 0.03, *n* = 8, Fig. 3E). Moreover, hemizygote patients showed age-dependent changes in flow heterogeneity of the splenium of the corpus callosum with peak values between 5–10 years of age (Fig. 3F). This association was confirmed by applying a random effects model (hemizygote subjects: β = −0.05185, 95% CI −0.093 to −0.010, *P* = 0.0176; controls: β = −0.00948, 95% CI −0.047 to 0.028, *P* = 0.6048, Fig. 3G and Model 2).

### Sequential increase in microvascular flow heterogeneity and blood–brain barrier permeability precede T_2_-weighted white matter abnormalities in CALD

**Figure 4:**
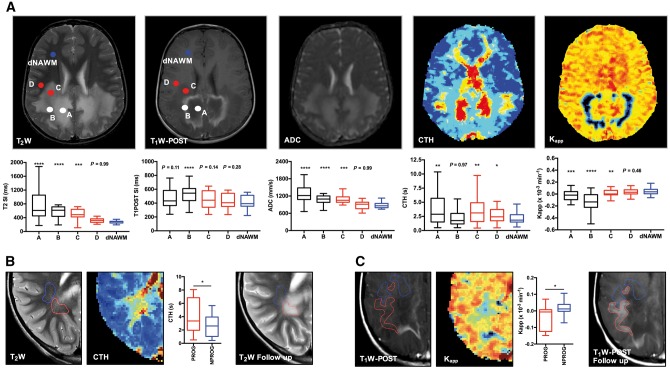
**Microvascular perfusion abnormalities precede T_2_-weighted abnormalities.** (**A**) CALD lesion segmentation into [A] T_2_-weighted (T_2_W) hyperintense necrotic core, [B] active inflammatory demyelination characterized by T_1_-weighted (T_1_W) imaging contrast enhancement; [C] the leading edge of the lesion (T_2_-weighted-hyperintense, non-enhancing on T_1_-weighted), adjacent [D] and dNAWM]and corresponding signal intensities for structural (T_2_-weighted, T_1_-weighted post-contrast), diffusion (ADC) and perfusion-based maps (CTH, K_app_) in such segmented regions. Data are presented as Tukey plots and are representative of 22 untreated individuals with progressive CALD. *P < 0.05, **P < 0.01, ***P < 0.001 and ****P < 0.0001. One-way ANOVA with repeated measures followed by Dunnett’s multiple comparisons test for CTH and K_app_ and Friedman test with Dunn’s corrections for multiple comparisons for T_2_-weighted, T_1_-weighted and ADC. (**B**) Representative T_2_-weighted and CTH maps of CALD illustrating areas of region D (adjacent NAWM) converting (red) versus non-converting (blue) to T_2_ hyperintensity on follow-up. Comparison of CTH signal intensities are presented as Tukey plots and are representative of nine untreated individuals with progressing CALD. *P < 0.05, two-tailed *t*-test. (**C**) Representative T_1_-weighted post contrast and K_app_ maps illustrating white matter in the outer rim of the CALD lesion progressing (red) versus a non-progressing (blue) to T_1_-weighted contrast enhancement on follow-up. Comparison of K_app_ signal intensities are presented as Tukey plots and representative of eight untreated individuals with progressive CALD. *P < 0.05, two-tailed *t*-test.

To determine if microvascular perfusion abnormalities can identify white matter at risk for disease progression once cerebral demyelination has ensued, we compared different perfusion variables in cerebral lesions segmented accordingly to their T_1_-weighted post-contrast and T_2_-weighted imaging characteristics (Fig. 4A). We found that perilesional NAWM (Zone D) had significantly higher CTH compared to dNAWM (3.01 ± 0.43 versus 2.20 ± 0.26, repeated measures ANOVA between-group difference *P* < 0.0001, *post hoc* Zone D versus dNAWM *P* = 0.024, *n* = 22) while no differences were found in structural integrity as indicated by apparent diffusion coefficient (ADC: 852 ± 47.44 mm/s versus 828.4 ± 43.54 mm/s, Friedman test between-group differences *P* < 0.0001, *post hoc* Zone D versus dNAWM, *P* = 0.999, Fig. 4A). When evaluating perilesional NAWM progressing to T_2_-weighted hyperintensity versus non-progressing NAWM we found significantly higher CTH (4.09 ± 0.87 s versus 2.62 ± 0.57 s, paired *t*-test, *P* = 0.0248, *n* = 9, Fig. 4B). Moreover, applying a linear mixed-model controlling for age, change in age, treatment and their interactions (Model 3) we confirmed that lesion volume increased over time at a higher rate in younger patients (*P* = 0.0137) and was associated with higher capillary flow heterogeneity in the perilesional NAWM on the previous scan (*P* = 0.0153).

Estimation of blood–brain barrier integrity by K_app_ demonstrated abnormal permeability beyond the T_1_-weighted contrast enhancing rim (Zone C) as compared to dNAWM indicating a gradual increase of blood–brain barrier permeability (0.01 ± 0.01 × 10^−3^/min versus 0.04 ± 0.01 × 10^−3^/min, repeated measures one-way ANOVA between-group difference *P* < 0.0001, *post hoc* Zone C versus dNAWM *P* = 0.0067, *n* = 22, Fig. 4A). K_app_ values were only elevated in those T_2_-weighted hyperintense regions that progressed to overt contrast enhancement on follow-up imaging (−0.04 ± 0.03 × 10^−3^/min versus 0.02 ± 0.02 × 10^−3^/min, paired *t*-test, *P* = 0.0118, *n* = 8, Fig. 4C).

**Figure 5:**
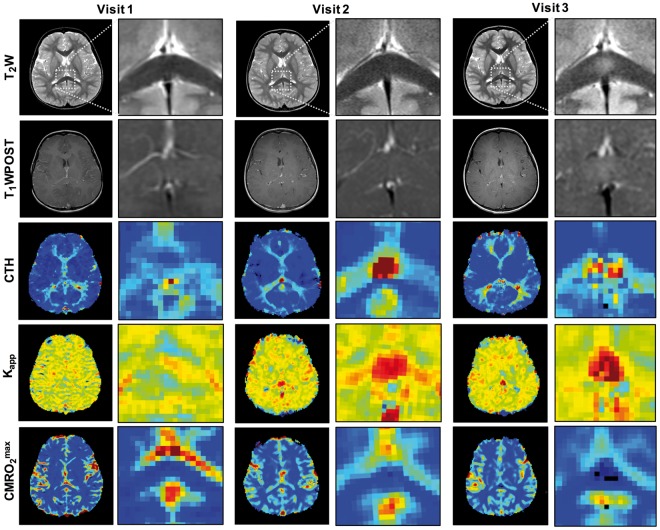
**Microvascular perfusion in a patient converting to cerebral disease.** Longitudinal sequential imaging in a young male hemizygote who converted to CALD. *Upper* two panels show structural T_2_-weighted (T_2_W) and T_1_-weighted post-contrast (T_1_WPOST) images. *Lower* panels show corresponding perfusion-based maps for each time point. Slightly elevated at the baseline visit, this patient developed a substantial peak in CTH and increased permeability (K_app_) within the splenium of the corpus callosum at the second visit 13 months later. While the T_2_-weighted- and T_1_-weighted post-contrast images showed no abnormalities at this time-point. Follow-up scan 6.5 months later revealed a corresponding T_2_-weighted-hyperintense region. Areas of increased CTH moved further outward from this lesion while blood–brain barrier permeability increased in the core. T_1_-weighted images show slight, hazy contrast enhancement. Low CMRO_2_^max^ coincides with appearance of the T_2_-weighted lesion. The subject underwent MRI serial screening due to confirmed hemizygote status, was successfully treated with HSCT and remained neurologically asymptomatic during the observation period.

In our study, of 10 hemizygotes followed with biannual MRIs for screening, three converted to cerebral disease within the observation period. Of these, two subjects developed a lesion within the corticospinal tracts and one within the splenium of the corpus callosum. Longitudinal sequential imaging in the latter showed a substantial peak in CTH and leakage parameter K_app_ within the SPL 6.5 months prior to the detection of T_2_-weighted abnormalities on conventional MRI. These flow abnormalities, although initially compensated by an increase in OEC, caused substantially compromised CMRO_2_^max^ by the time the lesion appeared on structural imaging, indicating failure to meet oxygen demand and possibly resulting in metabolic derailment (Fig. 5).

### Microvascular perfusion abnormalities normalize after haematopoietic stem cell transplantation

**Figure 6:**
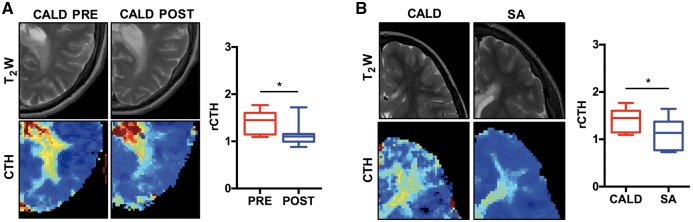
**Microvascular perfusion abnormalities normalize after HSCT.** (**A**) Representative baseline and follow-up T_2_-weighted (T_2_W) and perfusion-based CTH scans in a subject with CALD that underwent successful HSCT. Comparison of CTH signal intensity in NAWM adjacent to the CALD lesion at baseline and follow-up within the first year after HSCT (0.6 ± 0.3 years) is presented as Tukey plots of mean of nine treated individuals with CALD. *P ≤ 0.05, two-tailed *t*-test. (**B**) Representative baseline (T_2_-weighted and CTH) of a subject with progressing CALD (*left*) compared to a subject with self-arrested CALD (SA, *right*). Comparison of same regions as in (**A**) between progressive CALD and non-progressing CALD cases is presented as Tukey plots of means of 22 untreated CALD cases and 15 perfusion scans of three subjects with self-arrested CALD individuals with CALD. *P < 0.05, two-tailed *t*-test.

In subjects followed for at least 2 years after HSCT we found significant decrease in capillary flow heterogeneity in the outer rim of the T_2_-weighted hyperintense region as well as in the perilesional NAWM already within the first year (rCTH: 1.4 ± 0.07 versus 1.12 ± 0.06, unpaired *t*-test, *P* = 0.003, *n* = 10, Fig. 6A). Similarly, low values were measured in corresponding regions in a small subset of untreated CALD patients without lesion progression (self-arrested cases) (rCTH: 1.10 ± 0.09 versus 1.42 ± 0.07, unpaired *t*-test, *P* = 0.0157, *n* = 13 and *n* = 10, Fig. 6B).

## Discussion

Through application of a vascular model to dynamic susceptibility contrast magnetic resonance perfusion data, we demonstrated for the first time that ABCD1 dysfunction alters white matter microvascular physiology in ALD patients and that these disturbances in capillary flow precede T_2_-weighted abnormalities and contrast enhancement on structural MRI. Consistent with the hypothesis that increased brain endothelium–leucocyte interactions caused by ABCD1-related endothelial upregulation of adhesion molecules leads to alterations in blood flow across capillaries, we found higher CTH in NAWM of asymptomatic ALD hemizygote patients compared to controls (Fig. 2). Heterogeneity of flow across a capillary bed and cerebral blood flow are the two major determinants of oxygen availability to the brain parenchyma (Rasmussen *et al.*, 2015). During periods of increase metabolic demand, augmentation and/or homogenization of flow can secure adequate oxygen delivery and thus, failure to homogenize capillary flow (high CTH values) can result in reduced oxygen availability altering tissue homeostasis and viability (Jespersen and Østergaard, 2012). In male hemizygotes without cerebral disease we found that the increased flow heterogeneity, in spite of compensatory higher OEC, results in a reduced upper limit of metabolic rate of oxygen (CMRO_2_^max^) in the white matter and to a lesser extent in the grey matter (Fig. 2 and Supplementary Table 2). While raw perfusion data do not allow calculation of the exact amount of oxygen extraction into the tissue, a reduction on its maximum metabolic capacity, CMRO_2_^max^, indicates suboptimal tissue perfusion and suggests that capillary flow dysfunction contributes to white matter degeneration in ALD.

In contrast with other white matter inflammatory disorders, such as multiple sclerosis and acute demyelinating encephalomyelopathy, demyelinating lesions in CALD follow a striking stereotypical pattern with up to 80% starting in the splenium of the corpus callosum and subsequently spreading in a confluent manner around the ventricles (van Geel *et al.*, 2001; Loes *et al.*, 2003; Eichler *et al.*, 2007) (Fig. 1). To assess if this anatomical predilection is associated with a specific microvascular susceptibility to ABCD1 deficiency, we analysed perfusion parameters in hemizygote subjects across different white matter regions and compared to a statistical atlas of lesion frequency distribution of a cohort of CALD cases. We found that the splenium of the corpus callosum, the most frequently affected region, carries the highest heterogeneity of flow when compared to less frequently affected white matter areas such as the frontal white matter. Neuroanatomic studies of the brain vascular system indicate that blood supply patterns in white matter are regionally different, explaining the variations in vulnerability to perfusion or oxygen deficiency (Moody *et al.*, 1990). Notably, some of these perfusion abnormalities also coincide with the white matter regions where oligodendrocytes express highest levels of ABCD1 (corpus callosum, corticospinal tract and anterior commissure) (Fouquet *et al.*, 1997) and with higher vulnerability to hypoperfusion due to increased metabolic demand from large myelinated tracts depending on distal flow via long penetrating arterioles (Brown and Thore, 2011). Furthermore, applying a random effects model controlling for the effect of age upon CTH we found that increases in CTH are associated with an absolute increase in probability of demyelination (Fig. 3).

We then investigated if time affected white matter microvascular parameters by analysing regional changes in capillary flow, as measured by normalized CTH (rCTH) in the splenium of the corpus callosum of the first and last available scans in a small cohort of hemizygotes. If followed over time, CTH values in the splenium of the corpus callosum peaked between 5–10 years of age in ALD patients as compared to controls and while splenium of the corpus callosum rCTH was not significantly affected by age in our control cohort, we found a decrease with ageing in hemizygotes (Fig. 3). Strikingly, these temporal differences in flow abnormalities in hemizygote subjects concur with the period of brain development with highest metabolic demand (Kuzawa *et al.*, 2014) in a region with lower cerebral blood flow relative to the density of axonal packing (Giezendanner *et al.*, 2016) and greatest susceptibility for conversion to CALD (Eichler *et al.*, 2007; Moser *et al.*, 2007; Berger *et al.*, 2014), indicating a selective, age-dependent effect of the ABCD1 deficiency state upon the white matter regions most susceptible to cerebral demyelination.

ABCD1 deficiency is necessary but not sufficient to develop CALD. The factors that contribute to conversion to inflammatory demyelination are still unknown but early presence of contrast enhancement on MRI and CALD onset after brain contusion (Raymond *et al.*, 2010; Bouquet *et al.*, 2015) suggest that disruption of the blood–brain barrier may be a critical trigger. Our recent *in vitro* findings also suggest that ABCD1 deficiency impacts brain microvasculature in ways that may be critical not only to initiation but also to progression of the inflammatory demyelination (Musolino *et al.*, 2015). In this study, we confirmed that microvascular perfusion abnormalities can identify white matter at risk of disease progression once cerebral demyelination has ensued. Specifically, we found following lesion segmentation according to their T_1_-weighted-post contrast and T_2_-weighted imaging characteristics adapted from previous studies (Musolino *et al.*, 2012) that perilesional NAWM (Zone D) progressing to T_2_-weighted hyperintensity had significantly higher CTH compared to dNAWM and controls. Moreover, applying a linear mixed model we established that lesion volume growth was strongly associated with CTH values of perilesional NAWM on the previous scan. We also found that abnormal K_app_ precedes overt contrast enhancement and is an early biomarker of increased white matter blood–brain barrier permeability (Fig. 4).

Even though CTH and K_app_ have not yet been studied in other inflammatory demyelinating disorders, alterations in cerebral perfusion preceding overt blood–brain barrier permeability and structural white matter changes have been described suggesting that microvascular dysfunction is a common early marker of white matter inflammatory demyelination. In patients with optic neuritis, increased blood–brain barrier permeability in NAWM was associated with a higher risk of conversion to multiple sclerosis (Cramer *et al.*, 2015). In relapsing remitting multiple sclerosis, elevated perfusion was found a few weeks before the appearance of contrast enhancing lesions (Wuerfel *et al.*, 2004)*.* In contrast, in primary progressive multiple sclerosis and in Balo’s disease, which like CALD are characterized by their inherit inability to self-arrest and confluent expansion, hypoperfusion in perilesional NAWM has been described (Mowry *et al.*, 2007; Inglese *et al.*, 2008; Musolino *et al.*, 2012). These differences between relapsing and primary progressive inflammatory lesions may indicate that perfusion abnormalities correlate with the pattern of lesion onset and propagation.

These changes in microvascular flow dynamics may be attributed to multiple mechanisms including vasoreactivity to meet higher metabolic demand in areas of active inflammation (Østergaard *et al.*, 2014), impaired capillary flow caused by increased adhesion to leucocytes (Schmid-Schönbein *et al.*, 2001) and abnormal glial-endothelial interactions (Aubourg, 2015; Musolino *et al.*, 2015; Absinta *et al.*, 2016). For example, correlation of perfusion imaging and histopathology in a rat model of multiple sclerosis demonstrated that regions with increased perfusion correspond to areas with vessel dilatation but without marked leucocyte infiltration (Broom *et al.*, 2005), while perilesional NAWM with hypoperfusion in CALD coincides with perivascular microglia activation (Musolino *et al.*, 2015). Moreover, in CALD mitochondrial dysfunction and increased oxidative stress derived from accumulation of VLCFA due to loss of ABCD1 function (Fourcade *et al.*, 2008; Berger *et al.*, 2014) may contribute to the selective spatial and temporal microvascular flow deregulation we observed in NAWM of asymptomatic hemizygotes. Supporting this hypothesis, we found increased white matter CTH and changes in K_app_ several months prior to the detection of cerebral disease on conventional MRI. These flow abnormalities, although initially compensated by an increase in OEC, caused substantially compromised CMRO2^max^ by the time the lesion appeared on structural imaging, indicating failure to adjust to the oxygen demand, ultimately resulting in metabolic derailment and degeneration (Fig. 5).

Currently, the only approved treatment to arrest CALD is HSCT, presumably by bone marrow-derived monocytes with healthy ABCD1 that migrate into the brain and differentiate into less inflammatory macrophages and microglia (Rafii and Lyden, 2003; Yamada *et al.*, 2004; Mahmood *et al.*, 2007; Kemp *et al.*, 2016). The success of this effective but highly toxic treatment has been associated with disappearance of contrast enhancement and normalization of perfusion in T_2_-weighted hyperintense cerebral tissue (Musolino *et al.*, 2012; Miller *et al.*, 2016). Following subjects treated with HSCT, we found a significant decrease (normalization) in capillary flow heterogeneity in the outer rim of the T_2_-weighted hyperintense region as well as in the adjacent NAWM. Similarly, low values were measured in corresponding regions in a small subset of untreated CALD subjects without lesion progression (self-arrested, Fig. 6); a subgroup that makes up to 10–15% of all CALD cases (Korenke *et al.*, 1996). In the future, combining assessment of microvascular flow heterogeneity with diminishing contrast enhancement, may potentially allow identification of self-arresting cases, guide follow-up interval timing and prevent unnecessary procedures and interventions in this patient population.

Our study has several limitations including that contrast-based magnetic resonance perfusion imaging has limited spatial resolution and derived fitted residue functions for each voxel represent an average retention across hundreds of capillary beds. The contrast agent leakage term K_app_ is supposed to describe the flux of contrast agent from the intravascular space into the extravascular, extracellular space (Hansen *et al.*, 2017). However, the signal from each voxel consists of a combination of T_1_-weighted and T_2_*-weighted effects and intra- as well as extra-vascular compartments, which are not separable from a single gradient echo planar imaging DSC measurement (Hansen *et al.*, 2017). This limits the information that can be derived regarding quantification of blood–brain barrier permeability. While leakage terms are used to improve diagnostic accuracy of perfusion-based estimations of cerebral blood flow in diseases with impaired blood–brain barrier in the field of neuro-oncology (Emblem *et al.*, 2011), true physiological meanings of changes in K_app_ are yet to be established (Hansen *et al.*, 2017). Another limitation of our study is the inability to assess potential effects of general anaesthesia upon CTH, since sedation is necessary to scan young children and therefore strongly linked to age. To minimize potential confounders, we normalized these parameters, matched patients by age for group comparisons and confirmed that no significant haemodynamic changes occurred during anaesthesia. We also acknowledge that the changes in microvascular flow dynamics that we observed in the perfusion data are likely caused by a combination of multiple mechanisms.

Despite presenting the largest ALD magnetic resonance perfusion imaging cohort to date, the low prevalence of ALD limits the number of subjects available for the study and the positive predictive value of any biomarker. Several different statistical approaches were used to include additional available data whenever possible to increase confidence. Caution should be applied for the interpretation of the predictive value of CTH and K_app_ as our study is a single centre retrospective study prone to selection bias. Further the local perfusion abnormalities detected prior to conversion to CALD are based on one patient. Larger prospective multicentre studies of asymptomatic ALD hemizygote patients diagnosed by newborn screening will be necessary to validate DSC magnetic resonance perfusion imaging as a biomarker of early CALD.

In summary, we demonstrate *in vivo* that ABCD1 dysfunction affects white matter vascular physiology, strongly suggesting that microvascular flow abnormalities play a role in the pathophysiology of CALD. Our data imply that a sequence of increased capillary flow heterogeneity followed by an increase in blood–brain barrier permeability precede inflammatory demyelination in ALD. Although our study relies on a small cohort of patients we were able to demonstrate that ABCD1 deficiency causes perturbation in white matter perfusion during the developmental period and in the anatomical regions with highest susceptibility for conversion to cerebral disease. Elucidation of the exact mechanisms underlying these alternations in vascular physiology could lead to new and less toxic preventive or therapeutic approaches for CALD. We further show that DSC magnetic resonance perfusion may offer a powerful early biomarker for identification of patients at high-risk of conversion to cerebral disease and treatment response.

## Funding

Funding for this study was provided by the following awards from the National Institute of Neurological Disorders and Stroke (NINDS): K12NS066225 and K08NS52550.

## Supplementary material

Supplementary material is available at *Brain* online.

## Supplementary Material

Supplementary Tables

## Abbreviations

ALD: X-linked adrenoleukodystrophy
CALD: cerebral adrenoleukodystrophy
(d)NAWM: (distant) normal-appearing white matter
DSC: dynamic susceptibility contrast
HSCT: haematopoietic stem cell transplantation
OEC: oxygen extraction capacity
(r)CTH: (relative) capillary transit time heterogeneity
