# Supplementary material for: ABCD1 dysfunction alters white matter microvascular perfusion
Source: Brain. 2017 Nov 9;140(12):3139–52. doi: 10.1093/brain/awx262 (PMC5841142; doi:10.1093/brain/awx262)
Supplement: Supplementary Tables [file brain-2017-00511-file009_awx262.pdf]

<u>Supplementary tables:</u>

Supplementary Table 1: Male ALD perfusion imaging cohort

	CALD with perfusion imaging (n=33)	CALD follow up w/o HSCT (n=10)	CALD follow up post HSCT (n=9)	HEM (n=10)
**Age at baseline MRI scan:**	11.6 +- 9.4	7.69 +- 2.38	12.6 +- 10.1	6.1 +- 4.0
**Adults>20y, n (%)**	5 (15.2)	0 (0)	1 (11.1)	3 (30)
**Adolescence >10y<20y, n (%)**	5 (15.2)	1 (10)	2 (22.2)	3 (30)
**Children <10y, n (%)**	23 (69.7)	9 (90)	5 (55.5)	4 (40)
**Self-arrested, n (%)**	5 (15.2)	-	-	-
**Excluded*, n (%)**	2 (6.1)	-	-	-
**Conversion HEM to CALD, n (%)**	-	-	-	3 (30.0)
**MRI field strength MRIs:**				
**1.5T, n (%)**	21 (19.0)	10 (45.5)	4 (22.2)	23 (42.6)
**3T, n (%)**	89 (80.9)	12 (54.5)	14 (77.8)	31 (57.4)
**Total**	110	22	18	54
**Lesion location:**				
**Posterior, n (%)**	24 (72.7)	10 (90.9)	8 (88.9)	-
**Anterior, n (%)**	4 (12.1)	1 (9.1)	1 (11.1)	-
**Posterior and anterior, n (%)**	1 (3.0)	0 (0)	0 (0)	-
**Infratentorial, n (%)**	2 (6.1)	0 (0)	0 (0)	-
**Corticospinal tract, n (%)**	2 (6.1)	0 (0)	0 (0)	-
**Baseline Loes Score:**	3.25 (2.0-9.0)	9.0 (1.0-18.0)	9.0 (2.0-13.75)	-
**Baseline T2W lesion Volume, cc median (interquartile range)**	10.05 (4.1-20.4)	40.3 (0.9-168.5)	17.2 (4.4-74.4)	-
**Follow up Loes**	-	11.0 (3.0-23.0)	11.0 (2.5-17.0)	-
**Follow up T2W lesion volume, cc median (interquartile range)**	-	47.6 (5.0-204.5)	25.2 (4.7-111.5)	-
**Interval to follow up, years (SD)**	-	0.6 +- 0.4	0.8 +- 0.3	-
**Time post HSCT**	-	-	0.6 +- 0.3	-

Supplementary Table 1: Demographic and imaging characteristics of the male ALD perfusion cohort. Abbreviations: ALD= X-linked adrenoleukodystrophy; CALD = Cerebral ALD; HSCT= haematopoetic stem cell transplantation; HEM = male ALD subjects without

cerebral disease; * two patients were excluded due to corrupted perfusion data; T2W= T2-weighted.

Supplementary Table 2: Comparison of white matter and grey matter perfusion

	CON (n=10)	HEM (n=10)
**Age at MRI, mean years (range):**		
**Adults>20y, mean y (range)**	52.55 (51-53)	51.4 (50-52)
**Adolescence >10y<20y, mean y (range)**	14.66 (13-16)	13.94 (13-19)
**Children <10y, mean y (range)**	3.28 (3-4)	4.56 (3-6)
**Male sex, n (%)**	10 (100)	10 (100)
**MRI field strength:**		
**1.5T, n (%)**	6 (60)	6 (60)
**3T, n (%)**	4 (40)	4 (40)
**General anesthesia, n (%)**	4 (40)	4 (40)
**- IV Catheter 22G, n (%)**	4 (100)	3 (75)
**- CO_2 exp mmHg, mean (range)***	40.25 (26-48)	39.75 (36-48)
**- SBP mmHg, mean (range)***	85.25 (69-100)	94 (84-103)
**- DBP mmHg, mean (range)***	40.75 (33-46)	40.75 (35-45)
**- Pulse bpm, mean (range)***	99 (87-108)	89 (84-99)
**- % SpO2, mean (range)***	99 (98-100)	98.75 (98-100)
**- RR (Insp/min)***	26.25 (18-32)	21 (12-34)

Supplementary Table 2: Comparison of white matter and grey matter perfusion. Abbreviations: CON = controls; HEM = male ALD subjects without cerebral disease; BPS = blood pressure systolic; BPD = blood pressure diastolic; bpm = beats per minute; RR = respiratory rate.
